# Forearm pressure distribution during ambulation with elbow crutches: a cross-sectional study

**DOI:** 10.1186/1743-0003-11-61

**Published:** 2014-04-15

**Authors:** Jonas Fischer, Corina Nüesch, Beat Göpfert, Annegret Mündermann, Victor Valderrabano, Thomas Hügle

**Affiliations:** 1 Osteoarthritis Research Center, University Hospital Basel, Spitalstrasse 21, 4031 Basel, Switzerland; 2 Laboratory of Biomechanics and Biocalorimetry, Clinical Morphology and Biomedical Engineering, University of Basel, Basel, Switzerland; 3 Division of Sport Science, University of Konstanz, Konstanz, Germany

**Keywords:** Crutch gait, Forearm, Pressure distribution, Peak pressure, Center of pressure

## Abstract

**Background:**

Increasing numbers of patients require permanent walking aids to maintain mobility. Current elbow crutches are not designed for long-term use, and overuse is often associated with hematoma formation and pain along the forearm. We therefore hypothesized that the highest pressures between the forearm and crutch cuff during walking and stance are located in the ulnar region and that the level of weight-bearing, forearm circumference and kinematic parameters influence peak pressure values and pressure distribution.

**Methods:**

Ten healthy adults participated in a cross-sectional study. A pressure sensor array was attached to the forearm of each participant separating the forearm into four quadrants (lateral, ulnar, intermediate and medial). Measurements were taken during crutch gait and during partial and full weight-bearing stance. A three-dimensional motion analysis system with reflective markers attached to the subject’s body and to the crutches was used to obtain kinematic data.

**Results:**

The mean pressure on the forearm during crutch gait was 37.5 kPa (SD 8.8 kPa). Highest mean pressure values were measured in the ulnar (41.0 kPa, SD 9.6 kPa) and intermediate (38.0 kPa, SD 9.0 kPa) quadrants. The center of pressure was mainly located in an oblique lamellar area in these two quadrants. With increasing weight-bearing on the crutches during stance, we observed a shift of the peak pressures towards the ulnar quadrant. The circumference of the forearm correlated with the peak pressure in the medial and intermediate quadrants during crutch gait (P < 0.05). Peak pressures on the forearm showed a trend towards correlation with crutch abduction, but no association with other kinematic parameters was detected.

**Conclusion:**

The pressure load on the forearm during crutch-assisted gait is located predominantly over the ulna and may be linked to a range of secondary conditions caused by crutch use including hematoma formation and pain.

## Introduction

As the number of people living to an advanced age increases, the number of individuals suffering from degenerative diseases such as osteoarthritis (OA), spinal stenosis or disability also increases [1]. This development is reflected by an increase in patients requiring and benefitting from permanent walking aids in form of crutches or walkers for maintaining mobility. For instance, the use of walking aids significantly improves the quality of life in patients with knee OA [2].

However, crutch-assisted walking costs twice the energy of normal gait [3,4] and induces greater loads on the upper extremities; the glenohumeral joint may be loaded by more than 100% body weight during crutch gait [5]. The use of elbow crutches can trigger tenosynovitis in the biceps tendon [6] and cause ulnar neuropraxia at the wrist [7] and at the forearm [8]. Clinical experience shows that patients using elbow crutches may suffer from pain or skin hematoma, notably along the ulnar bone. In more severe circumstances, cases of ulnar bone fracture during crutch gait have been reported [9,10].

We speculated that these symptoms are related to excessive pressures applied to the forearm by the crutch cuff and that these pressures will be affected by the crutch position relative to the body. Understanding the parameters affecting the mechanical interface between the forearm and the crutch are critical for designing improved crutches. Sala et al. [11] have shown that crutch handle design influences palmar pressure distribution, reduces pressure loads in specific anatomic regions and increases the load-bearing area on the palmar surface of the hands during ambulation. However, to date information on the topographic pressure distribution between the forearm and the crutch cuff during crutch-assisted walking and stance is not available.

The purpose of this study was to test the hypotheses that the highest pressure between the forearm and crutch cuff during walking and stance is located in the ulnar region and that the level of weight-bearing, forearm circumference and kinematic parameters influence peak pressure values and pressure distribution.

## Methods

A homogenous group of ten healthy male volunteers participated in this study after providing informed consent. The study was conducted in accordance with the declaration of Helsinki. The patients’ mean age was 26.6 years (range 23 to 38 years) and their mean body mass index (BMI) was 24.1 kg/m^2^ (range 21.9 to 26.8 kg/m^2^). None of the patients had any orthopedic injuries within the preceding twelve months. Three participants had previously used crutches because of an injury. An experienced physiotherapist ensured the correct crutch configuration and position (Rebotec, Quakenbrück, Germany). The length from the olecranon to the styloid process of the ulna and the circumference of the forearm at the level of the proximal end of the cuff were measured using a tape measure.

### Pressure measurement

Pressure distributions were measured using a pressure sensor array (Sensor Model 5101; Tekscan Inc., South Boston, USA; 15.5 sensel per cm^2^; 120 frames per second). Prior to each data collection session, the sensor arrays were calibrated using a two-point calibration procedure and by applying a known static weight according to the manufacturer guidelines. While the reliability of the Tekscan system for measuring pressures between the crutch cuff and forearm were not available, intra class correlation coefficients for plantar pressure measurements during gait using this system reflected good to moderate reliability [12].

**Figure 1:**
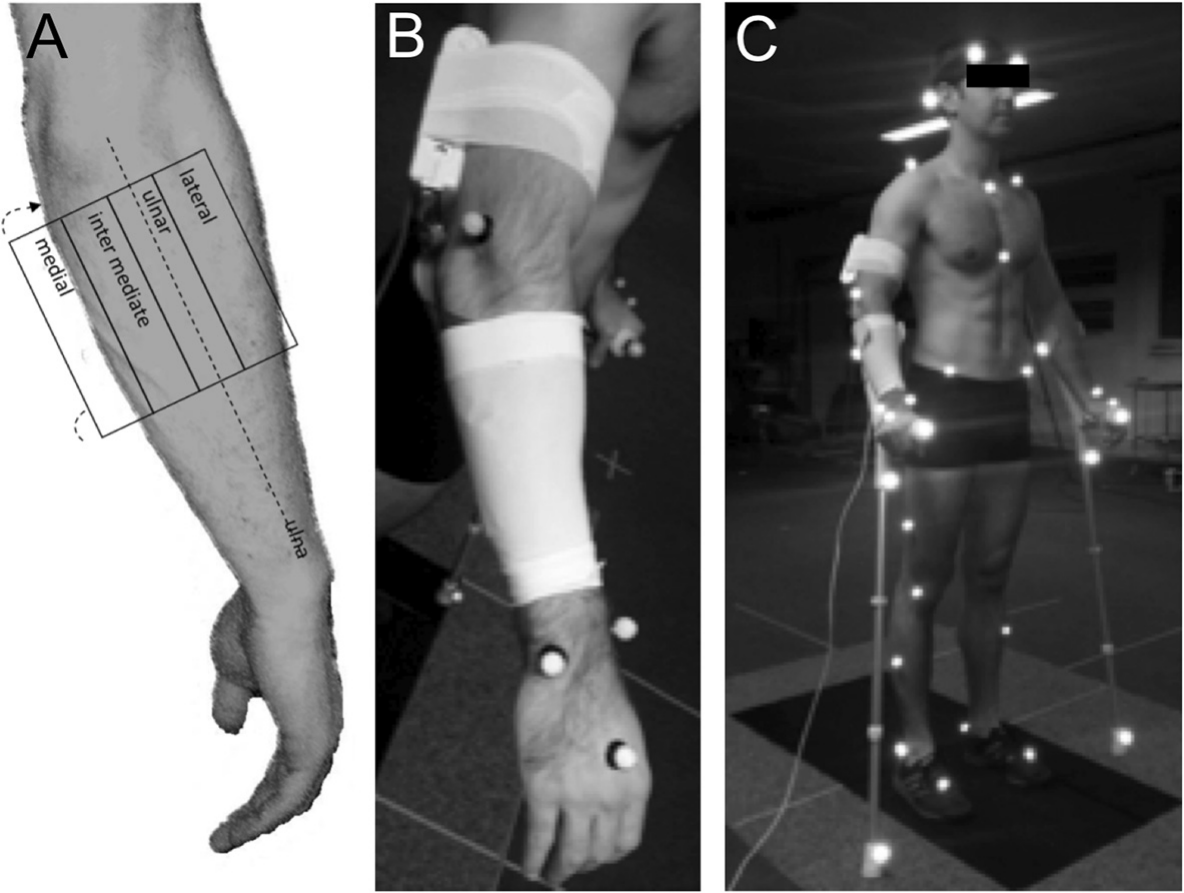
**Experimental set-up. A**. Mask of forearm. **B**. Sensor positioning. **C**. Marker set-up.

The subject’s right ulna was palpated, and the pressure sensor array was positioned following a mask dividing the forearm into four quadrants, medial, intermediate, ulnar and lateral. Each region measured 11.2 cm × 2.8 cm (Figure 1A). The sensor was secured to the forearm using an adapted support stocking so that the end of the sensor array was aligned with the level of the proximal end of the cuff (Figures 1B and C). The sensor array was attached to a scanning electronics box that was connected to a computer via a 4.5 m USB cable. The box was attached to the upper arm using self-adhesive tape (Figure 1B). After every two participants, the sensor was replaced.

### Crutch gait analysis

A six-camera three-dimensional motion capture system (Vicon MX13+, Oxford, United Kingdom) and two force plates (Kistler, Winterthur, Switzerland) were used to capture kinematic data and to control weight-bearing. Reflective skin markers were attached to predefined anatomical landmarks and placed according to the Helen Hayes model for the lower body [13] and the upper body Plug In Gait model described by Gutierrez et al. [14]. Four additional markers were placed on each crutch, namely at the bottom, below the handle, at the tip of the handle and on the cuff (Figure 1C). Before collecting gait or stance data, a static calibration trial in neutral stance position was recorded.

### Experimental conditions

Subjects were given sufficient time to familiarize with the following four experimental conditions.

#### Normal crutch gait

The standard walking procedure was determined as: advance the crutches together with the right lower extremity, then advance the left lower extremity while bearing the weight on the crutches and the right lower extremity. Data for five crutch gait trials with at least three right forearm loadings each were recorded. Partial weight-bearing was controlled during all trials using the force plate data.

#### Partial weight-bearing stance

Participants performed two partial weight-bearing stance experiments with 50% and 75% body weight on the crutches, respectively. Participants stood on the force plate on their right foot with part of their body weight supported by their foot and the other part of their body weight supported by the crutches.

#### Balancing on the crutches

To simulate loading of the crutches with full body weight, participants were asked to balance on their crutches while lifting both feet off the ground. Data for as many attempts as possible were recorded during two 7-second trials.

### Data processing

For each experiment and participant, the recorded forearm pressure values were used to calculate the average and peak pressures for each quadrant and for the entire sensor. The center of pressure was calculated for each frame, and the path of the center of pressure was obtained for each trial. In addition, the pressure data within each region was summed for visualizing total pressure load.

The motion data was used to calculate flexion, adduction and rotation of the right shoulder and the flexion of the right elbow and the angles between the crutch and the upper body axis (“crutch abduction”) and between the crutch handle and the frontal axis of the pelvis (“handle rotation”) during the stance phase of the right lower extremity for each trial.

The peak pressure for most trials occurred at 50% of the stance phase of the right lower extremity and we hence related pressure values to kinematic parameters at that time point.

### Statistical analysis

All statistical tests were carried out in GraphPad Prism 5.02 (GraphPad Software Inc., USA). Data are represented as means ± one standard deviation. Significant differences between conditions were detected using a one-way analysis of variance (ANOVA) with forearm circumference as between subject factor and level of weight-bearing as within subject factor followed by Bonferroni’s Multiple Comparison test. Significant correlations between kinematic parameters and peak pressures were detected using Pearson correlation (GraphPad Prism 5.02). The significance level for all statistical tests was set *a priori* to 0.05.

## Results

### Mean pressure and load-bearing area

Biometric characteristics, mean pressure of the whole sensor and the size of the load-bearing area for each participant and experimental condition are presented in Table 1. The mean pressure during crutch gait ranged from 22.5 to 55.2 kPa and the mean loading surface from 20.5 to 65.7 cm^2^. Mean pressures during crutch gait were similar to those observed during 50% weight-bearing stance, and the mean size of the load-bearing surface during crutch gait was similar to that for 75% weight-bearing stance (Table 1). The area of the pressure-loaded area for the 75% and full weight-bearing conditions was more than three-fold that for the 50% weight-bearing conditions.

**Table 1:** Biometric data, mean pressure and size of the loaded forearm area

***Biometric data***				***Crutch walking***		***Stance 50% BW***		***Stance 75% BW***		***Stance 100% BW***	
***No.***	***Height [cm]***	***BMI [kg/m***^***2***^ ***]***	**CF *[cm]***	***MP***	***MLS***	***MP***	***MLS***	***MP***	***MLS***	***MP***	***MLS***
1	177	21.90	29	32.5	54.3	33.9	36.0	41.1	56.0	47.4	56.0
2	179	26.69	29	36.2	65.7	41.5	41.7	57.2	83.1	30.7	83.1
3	184	23.63	30	45.8	46.1	41.9	21.9	47.5	74.0	65.8	74.0
4	183	23.29	27	31.8	40.6	31.5	20.7	36.5	54.1	37.9	54.1
5	172	25.88	28	32.4	57.8	30.3	33.3	40.8	67.6	47.2	67.6
6	176	26.79	28.5	36.8	33.1	35.2	12.1	42.8	59.2	55.2	59.2
7	178	23.22	28	55.2	20.5	55.3	15.0	62.6	53.3	74.8	53.3
8	183	23.46	29	46.0	42.3	50.4	5.9	55.1	78.0	61.4	78.0
9	169	22.06	26.5	35.4	32.2	43.5	10.9	41.3	67.5	52.0	67.5
10	177	23.30	28	22.5	39.0	21.5	6.8	24.0	71.1	43.1	71.1
Mean	177.8	24.0	28.3	37.5	43.2	38.5	20.4	44.9	66.4	51.6	66.4
1SD	4.8	1.8	1.0	9.2	13.4	10.0	12.7	11.2	10.4	13.3	10.4

Data are shown for crutch walking and stance with 50%, 75% and 100% body weight (BW) supported by the crutches for all 10 subjects. The load distribution between the crutches and the subjects’ foot was controlled using two force plates. For the 100% BW stance, subjects were asked to balance on their crutches with both legs lifted off the ground.

*BMI* body mass index; CF forearm circumference; *MP* mean pressure [kPa], *MLS* size of the pressure loaded forearm area [cm^2^].

Mean pressures in the quadrants increased from crutch gait and the 50% weight-bearing stance to the 75% and full weight-bearing stance conditions (Table 2). Differences in peak pressure between the ulnar and the lateral quadrant were significant for 75% weight-bearing stance (p < 0.01) and full body-weight stance (p < 0.05).

**Table 2:** Mean (standard deviation) pressure (kPa) of the sensor quadrants

***Sensor quadrant***	***Crutch walking***	***Stance***		
		***50% body weight***	***75% body weight***	***Full body weight***
Lateral	35.4 (10.4)	35.2 (9.2)	35.9 (9.2)	38.9 (10.1)
Ulnar	41.3 (9.6)	42.6 (10.2)	55.5 (12.6)	60.2 (13.2)
Intermediate	38.1 (9.0)	36.8 (9.8)	45.4 (14.7)	58.9 (17.7)
Medial	34.8 (8.6)	34.6 (9.5)	39.2 (10.9)	47.7 (9.1)

### Location of peak pressures and center of pressure

**Figure 2:**
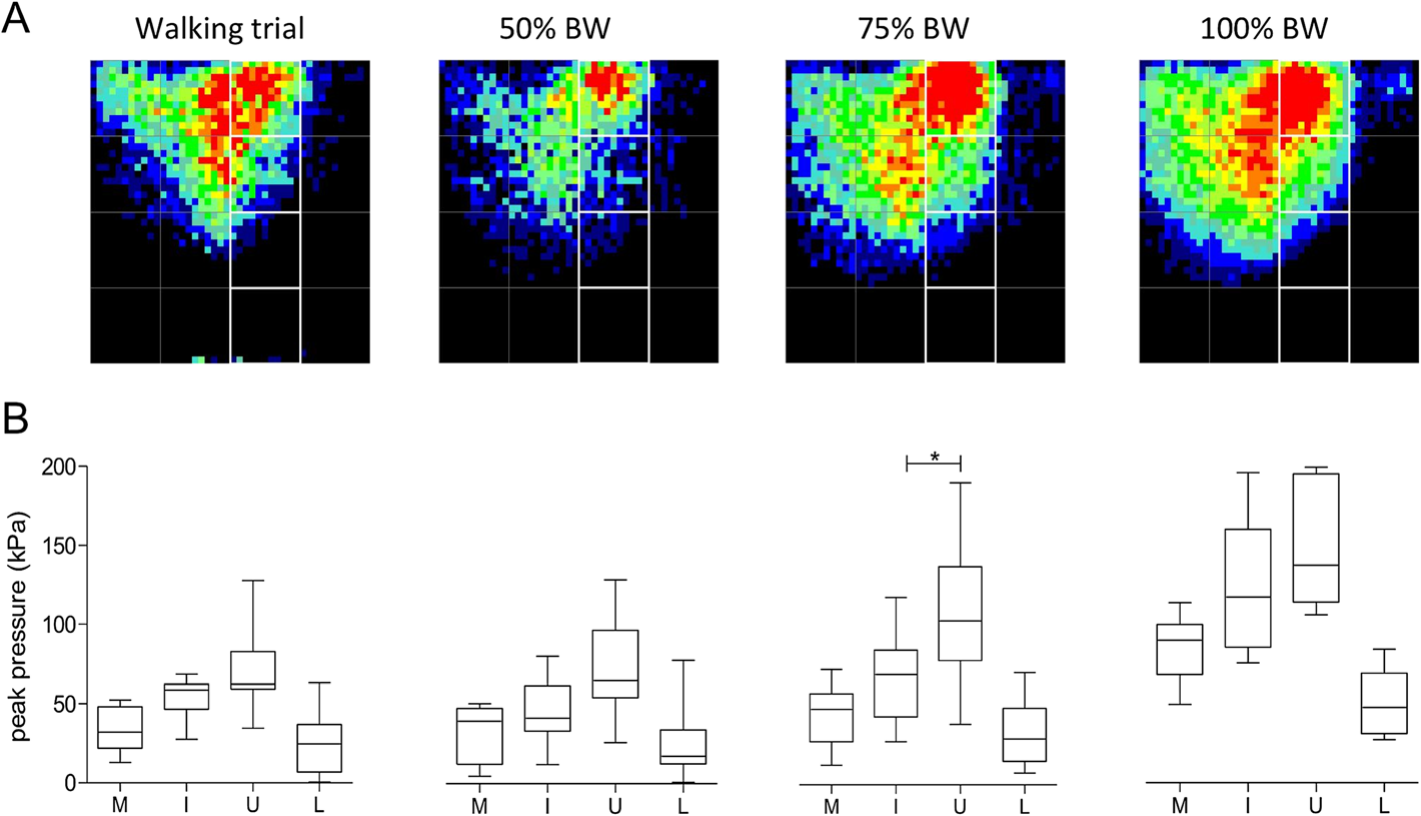
**Pressure outcome. A**: Total pressure distribution of all volunteers for each test (black: no pressure, red: high pressure). **B**: Averaged peak pressure values of all volunteers for each quadrant of the sensor (M: medial, I: intermediate, U: ulnar, L: lateral).

The summed total pressures were distributed in an oblique triangle shape located in the proximal zone of the intermediate and ulnar quadrants during crutch gait and broader in the ulnar quadrant during the stance experiments (Figure 2A). For most trials, the peak pressures were located in these two regions with the majority being observed in the proximal ulnar quadrant: for crutch gait, 50% weight-bearing and full weight-bearing stance, the peak pressure was in the ulnar region for seven participants and in the intermediate region for three participants; for 75% weight-bearing stance, the peak pressure was in the ulnar part for eight participants and in the intermediate region for two participants. Peak pressure values were similar for the intermediate and the ulnar quadrant for crutch gait and full weight-bearing and higher in the ulnar than the intermediate region for 50% and 75% weight-bearing stance (Figure 2B). The difference between the two quadrants was only statistically significant for the 75% weight-bearing stance condition (p < 0.05). Across all experiments, more than 75% of the total pressure load was found in the ulnar and the intermediate quadrant (Figure 2B).

**Figure 3:**
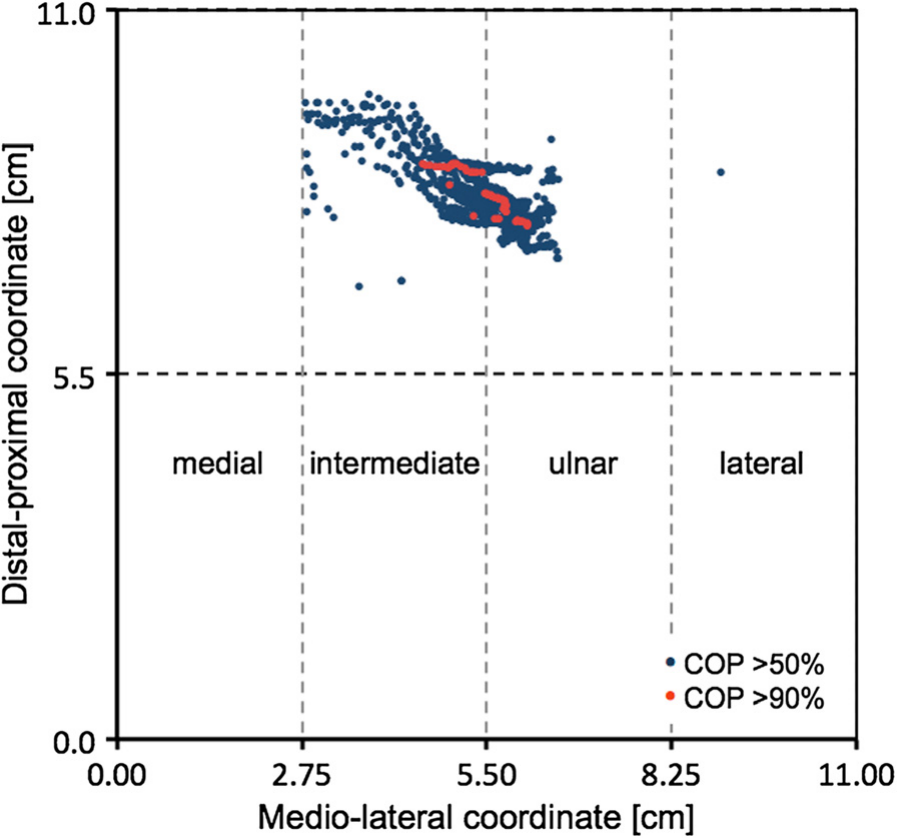
**Center of pressure.** Distribution of the centers of pressure (COP) during the loading phase above 50% (blue) and above 90% (pink) of the highest pressure value. The graphs show the 11 cm × 11 cm sensor for one volunteer.

The center of pressure was located in the proximal intermediate and ulnar quadrants (Figure 3). In four participants, the center of pressure moved little during walking, while in seven volunteers the center of pressure moved within an oblique lamellar area (Figure 3) shifting from the intermediate to the ulnar quadrant.

### Kinematic data

**Figure 4:**
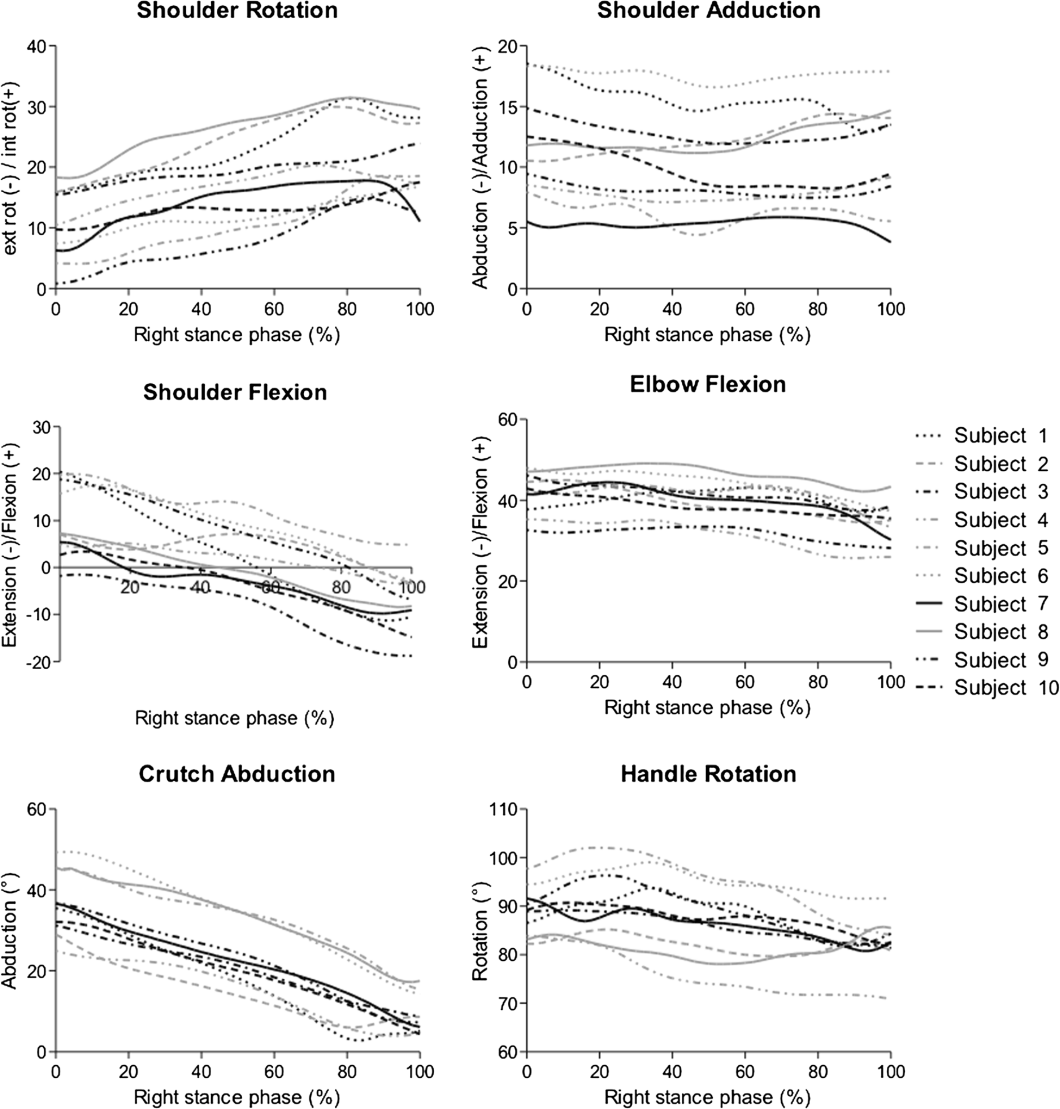
Angular kinematics during the stance phase of crutch gait for the right lower extremity.

Kinematics during the stance phase of crutch gait showed similar patterns for all participants: they walked with internally rotated shoulders, which increased towards the end of the stance phase, and with internally rotated crutch handles. While the shoulders were adducted throughout the stance phase, the crutches were abducted with the tips pointing outward. This crutch abduction decreased from the beginning to the end of the stance phase (Figure 4). For the partial and full weight-bearing conditions, all participants adopted the same position of slightly internally rotated and adducted shoulders and abducted crutches (Table 3).

**Table 3:** Mean (standard deviation) angular kinematics recorded during the stance trials for the right lower extremity

***Angle [°]***	***50% body weight***	***75% body weight***	***Full body weight***
Elbow flexion	42.6 (4.6)	43.9 (4.9)	45.7 (5.5)
Shoulder flexion	1.8 (8.2)	1.6 (9.8)	4.6 (5.5)
Shoulder adduction	12.1 (4.3)	10.9 (3.8)	13.2 (5.6)
Shoulder rotation	17.6 (6.5)	15.7 (6.0)	16.1 (5.6)
Crutch abduction	26.8 (7.9)	27.6 (9.1)	30.2 (7.2)
Handle rotation	81.9 (7.1)	80.7 (7.5)	81.7 (6.6)

### Relationship between peak pressure and kinematic and biometric parameters

**Figure 5:**
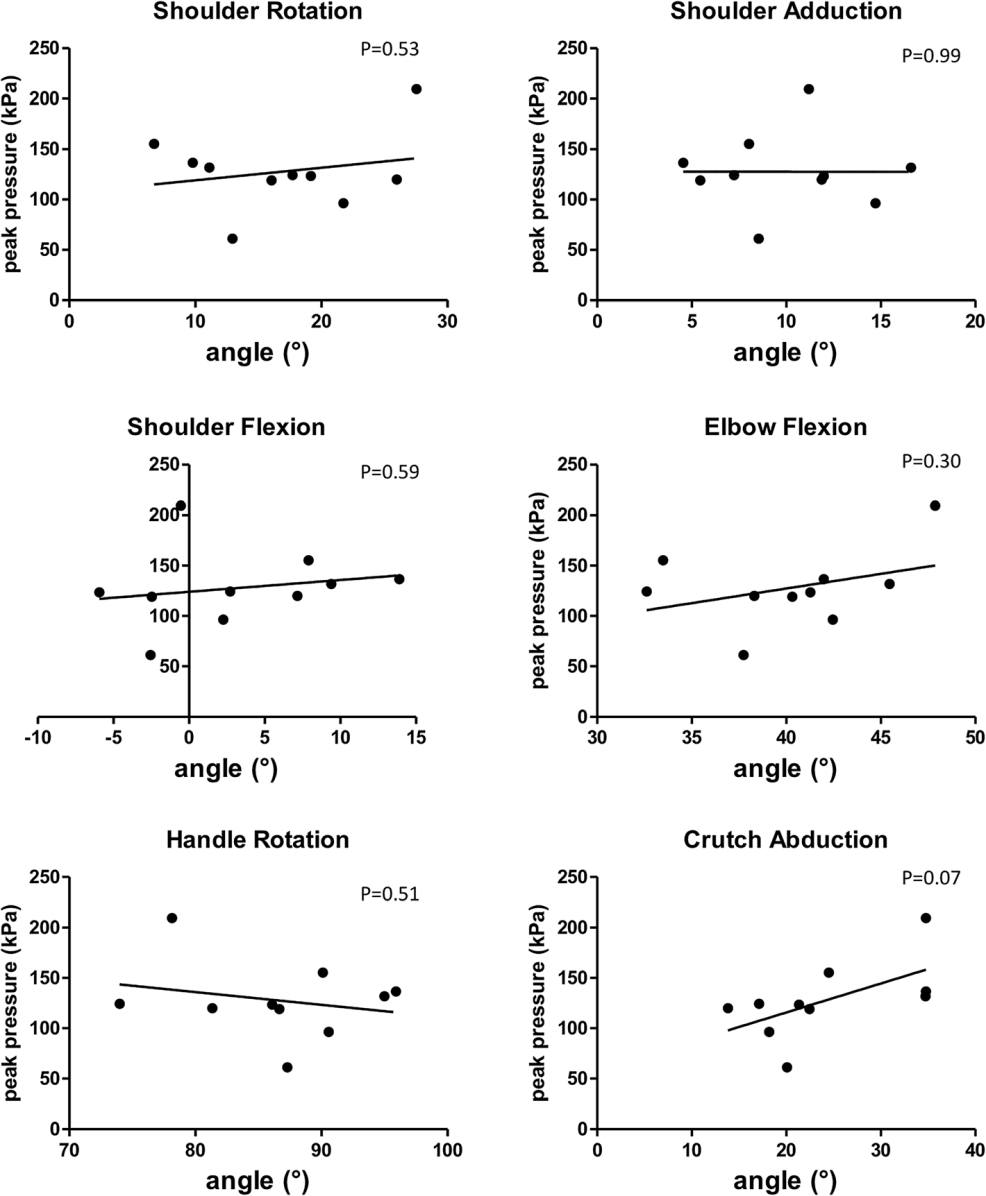
Correlation between kinematic parameters at 50% of the stance phase and the mean of the highest pressure values during crutch gait.

There was no significant correlation between peak pressure and any of the kinematic parameters at 50% of the stance phase during walking (Figure 5). However, there was a tendency towards higher peak pressures with greater crutch abduction (p = 0.07). The peak pressure in the intermediate (r = 0.658, p = 0.039) and the medial (r = 0.652, p = 0.041) quadrant significantly correlated with the circumference of the forearm. In addition, the peak pressure in the medial quadrant significantly correlated with the size of the load-bearing surface (r = 0.723, p = 0.018). Except for the significant correlation between peak pressure in the medial quadrant and the forearm circumference (r = 0.671, p = 0.034) during 50% weight-bearing, the correlations between peak pressure and both forearm circumference and size of the load-bearing area were stronger for the walking than for the stance conditions.

## Discussion

We found that the highest pressure loads during crutch gait were located over the ulna and mean pressures increased with increasing weight-bearing. Furthermore, the size of the pressure-loaded area for walking was twice that for the 50% load-bearing condition and half that for the 75% and full weight-bearing conditions. We observed peak pressures between 100 and 170 kPa for most volunteers, which were lower than the pressure values of 240 kPa on the palm during crutch gait reported by Sala et al. [11]. The high peak pressure loads applied to the palm are likely dispersed by the thicker soft tissue which serves to protect the underlying bones. The soft tissue covering the ulna is much thinner than that of the palm, and hence even peak pressure loads within the range measured in our study applied during extensive crutch walking may be sufficient for causing pain or hematoma along the ulnar bone as clinically observed. While we did not measure palmar pressure distributions in this study, it is possible that forces and moments applied to the crutch handle and those applied to the crutch cuff may counteract each other. Understanding this interaction is critical for avoiding that the problem shifts from one region to another when new crutch designs are introduced.

The highest average and maximum pressures as well as the centers of pressure were found in the intermediate and the ulnar quadrants; specifically, they were located in the proximal part for most participants. We found that—while with increasing load the center of pressure moved horizontally from the intermediate quadrant towards the ulnar quadrant—the size of the pressure-loaded area did not change. This horizontal shift of the crutch relative to the forearm may represent shear loads on the skin overlying the ulna, which may induce injuries such as those observed in people after prolonged crutch use [6-10]. In this study, we measured pressure but not frictional forces between the crutch cuff and the forearm. Hence, the data obtained in this study is not sufficient for testing this mechanism.

The correct adjustment of the crutches and kinematic patterns presumably are important parameters in minimizing and optimally distributing the loading on patients’ forearms. Of all kinematic parameters assessed in this study, only crutch abduction was weakly associated with higher peak pressures: the more abducted the crutches were the higher was the pressure. However, we only measured healthy persons with correctly adjusted crutches and did not provide instructions on how to hold them. Nevertheless, the kinematic patterns for the shoulder, elbow and crutch are in agreement with those reported previously [15] for one female subject without any previous experience in crutch-walking. Data for one step in our study roughly corresponded to 0 to 50% of an entire gait cycle reported by Bhagchandani et al. [15]. Although healthy subjects were tested in both studies, the agreement in kinematic data between these studies supports the validity and relevance of the data. In patients with knee osteoarthritis, cane use causes an immediate increase in energy expenditure and decrease in pain during gait [2]. Hence, an optimal crutch-gait pattern in patients will be presumably defined primarily by pain in their index joint or limb and by their energy expenditure and only to a lesser extent by the pressure loads between the crutch cuff and forearm. Moreover, walking with reduced crutch abduction may efficiently reduce pressure loads on the ulna in patients with ulnar pain during prolonged crutch walking. A comparison between healthy subjects and groups of patients and between different ambulation patterns is needed to better understand the association between kinematic patterns and pressure load between the crutch cuffs and the forearms.

The significant correlation between the circumference of the forearm and the medial side of the sensor during crutch gait indicates a different pressure distribution in bigger forearms. The relationship between forearm circumference and pressure distribution was more pronounced for walking than for standing. This result is particularly interesting because people more frequently perform walking than standing tasks while using crutches. However, our subjects had a relatively small range in forearm circumference (26.5 to 30.0 cm) and hence this relationship should be confirmed in subjects groups with larger variability in forearm circumferences.

Although there was a large range in the magnitudes of kinematic parameters during crutch gait and different pressure distribution patterns among our subjects, peak pressures were mainly found within the same oblique, triangle-shaped area. The centers of pressure of the participants—all found in this area—were clustered even closer together: they were either in a narrow strip or assembled in a small point over the ulna. Therefore, we propose that the pressure over the ulna could be reduced by a novel cuff design. The novel cuff shape should better distribute the pressure over the entire forearm, ideally to areas with more soft tissue, which presumably absorb pressure better than areas with little soft tissue. Furthermore, additional cuff padding should adopt the shape of the forearm anatomy and simultaneously reduce rubbing of the skin while ensuring stability and maneuverability. Finally, the new crutch design should account for differences in forearm circumference.

Patients who experience high pressure loading between the crutch cuff and the patient’s forearm may also adopt gait patterns in an attempt to relieve associated pain. Such altered gait patterns combined with pathologic conditions and/or altered shape of the humeral head may result in abnormal stress distributions in other structures such as the scapula as observed in a theoretical study by Büchler et al. [16]. Moreover, the amount of forearm rotation influences the anatomical regions that transfers loads between the ulna and the radius. Ishi et al. [17] showed in a cadaver study that in pronation the pressure load was concentrated in the dorsal portion of the sigmoid notch and in supination the pressure load was distributed on the palmar portion of the radioulnar joint. Hence, not only the interaction between the crutch and the body but also the orientation and motion of body segments during crutch gait are relevant for understanding the mechanical consequences on upper extremity joints.

The two measurement systems, Tekscan and Vicon, could not be synchronized, and we thus only related the gait parameters at 50% of the stance phase—which coincided with the highest pressure values—to the pressure parameters. Future study should relate kinematic data of the entire gait cycle to pressure distribution patterns between the crutch cuff and the forearm. Measuring the pressure distribution between the crutch cuff and the forearm only on the right side did not allow for a comparison between both sides. It is possible that the load is unevenly distributed between both crutches, which we did not control in this study. We used the Plug In Gait Model for calculating kinematic parameters, which has been previously used for studying upper body kinematics and kinetics [14,18]. However, this model does not exactly match the model recommended by the International Society of Biomechanics [19]. The primary aim of our study was not to provide upper body kinematic data of crutch gait but rather to relate differences in upper body kinematics to differences in cuff pressure distribution. The model recommended by the International Society of Biomechanics has been previously expanded to correct for compensating soft tissue artifacts in the upper-arm [20]. While some studies on crutch gait have used this model [15,21], others have applied other models [22]. Hence, there is a need for using standardized models when describing crutch gait in future studies. Finally, our study only involved healthy individuals and our results may differ from those of patients. Nevertheless, the data presented in this study contribute to a better understanding of crutch cuff-body interaction during ambulation.

## Conclusion

The pressure load on the forearm during crutch-assisted gait is located predominantly over the ulna and may be linked to a range of secondary conditions caused by crutch use. The circumference of the forearm influences the pressure distribution between the crutch cuff and forearm during walking and stance. The results of this study emphasize the need for new cuff designs and appropriate cuff sizing with the goal of preventing injuries in the ulnar region of the forearm, which presumably would have a profound impact on the healthcare system considering the large number of crutch users.

## Competing interests

The authors declare that they have no competing interests.

## Authors’ contributions

JF conceptualized the study and was responsible for data collection, processing, analysis and interpretation and drafted the manuscript; CN and BG assisted in data collection, processing and interpretation; AM was involved in data processing, analysis and interpretation and provided writing assistance; VV was involved in designing the study and data interpretation; TH conceptualized the study and contributed to data analysis and interpretation and manuscript preparation. All authors read and approved the final manuscript.
